# SDF-1alpha concentration dependent modulation of RhoA and Rac1 modifies breast cancer and stromal cells interaction

**DOI:** 10.1186/s12885-015-1556-7

**Published:** 2015-08-01

**Authors:** Jennifer Pasquier, Nadine Abu-Kaoud, Houari Abdesselem, Aisha Madani, Jessica Hoarau-Véchot, Hamda Al. Thawadi, Fabien Vidal, Bettina Couderc, Gilles Favre, Arash Rafii

**Affiliations:** 1 Stem Cell and Microenvironment Laboratory, Weill Cornell Medical College in Qatar, Education City, Qatar Foundation, Doha, Qatar; 2 Department of Genetic Medicine, Weill Cornell Medical College, New York, NY USA; 3 Department of Immunology and Microbiology, Weill Cornell Medical College in Qatar, Qatar Foundation, Education city, P.O. Box: 24144, Doha, Qatar; 4 EA 4553, Institut Claudius Regaud, Toulouse, France; 5 INSERM U1037 Cancer Research Center of Toulouse, Institut Claudius Regaud, Toulouse, France; 6 Department of Advanced gynecologic Surgery, Université Montpellier 1, Montpellier, France; 7 Department of Genetic Medicine and Obstetrics and Gynecology, Stem cell and microenvironment laboratory Weill Cornell Medical College in Qatar, Qatar-Foundation, PO: 24144, Doha, Qatar

**Keywords:** Breast cancer, Tumor microenvironment, Metastasis, SDF-1alpha, Stromal cells

## Abstract

**Background:**

The interaction of SDF-1alpha with its receptor CXCR4 plays a role in the occurrence of distant metastasis in many solid tumors. This interaction increases migration from primary sites as well as homing at distant sites.

**Methods:**

Here we investigated how SDF-1α could modulate both migration and adhesion of cancer cells through the modulation of RhoGTPases.

**Results:**

We show that different concentrations of SDF-1α modulate the balance of adhesion and migration in cancer cells. Increased migration was obtained at 50 and 100 ng/ml of SDF-1α; however migration was reduced at 200 ng/ml. The adhesion between breast cancer cells and BMHC was significantly increased by SDF-1α treatment at 200 ng/ml and reduced using a blocking monoclonal antibody against CXCR4. We showed that at low SDF-1α concentration, RhoA was activated and overexpressed, while at high concentration Rac1 was promoting SDF-1α mediating-cell adhesion.

**Conclusion:**

We conclude that SDF-1α concentration modulates migration and adhesion of breast cancer cells, by controlling expression and activation of RhoGTPases.

**Electronic supplementary material:**

The online version of this article (doi:10.1186/s12885-015-1556-7) contains supplementary material, which is available to authorized users.

## Background

Development of distant metastasis in breast cancer is responsible for the majority of cancer related deaths [1]. Metastasis happens through highly organized and organ specific sequential steps [2]. Among chemokines implicated in this cascade; SDF-1α/CXCR4 regulates organ specific colonization of metastatic tumor cells [3–6]. The stromal cell derived factor 1-α (SDF-1α) or CXCL-12 is physiologically expressed by mesenchymal stromal cells of metastasized breast cancer host organs such as liver, lungs, lymphatic tissues or bone marrow [7]. CXCR4 is over-expressed in many breast cancer cells (BCC), promoting cancer cell migration and invasion [8]. BCC differential chemokine receptor expression is correlated with their metastatic behavior [9]. CXCR4 expression predicts bone metastasis in breast cancer patients [10]. Two new ligands, the ubiquitin and the macrophage migration inhibitory factor were recently discovered to bind CXCR4, however their role in cancer biology has not been documented as much as SDF-1α [11–14].

Among many effects, SDF-1α/CXCR4 interaction regulates cancer cell motility and adhesion. [15]. Muller et al. showed that CXCR4 expression on breast cancers related to their migratory/metastatic behavior. They also illustrated that the inhibition of SDF-1α/CXCR4 interaction resulted in reduced metastasis in breast cancer xenograft models [16]. Concordantly, multiple studies showed that in different tumor types SDF-1α/CXCR4 interaction resulted in increased metastasis. SDF-1α signaling is involved in cell migratory properties, cell survival, homing and resistance to treatment [5, 17–19]. The mechanism through which SDF-1α can regulate such different proprieties as migration and adhesion (implicated in homing) is not clearly established.

It has been shown that CXCR4/SDF-1α interactions induced increased migration, proliferation and adhesion of breast cancer cells through different signaling pathways such as calcium mobilization [20], phosphorylation of src and fak [21], and phosphatidylinositol 3-kinase [22]. In multiple melanomas, SDF-1α increases homing, adhesion and invasiveness of cancer through the activation of GTPases of the Ras superfamily, RhoA and Rac1 [23]. Small GTPases play important roles in basic cellular processes such as cell proliferation, invasion, chemotaxis and adhesion [24]. Rho-protein-dependent cell signaling is important for malignant transformations [25]. RhoA activation triggers many pathways including Rho-associated protein kinase (ROCK) responsible for actin polymerization required for cell locomotion [26]. We have previously illustrated the role of Rho GTPases modulation in different neoplasic context such as melanoma, breast and ovarian cancers [27–31].

Here, we investigated the effect of different concentrations of SDF-1α in the modulation of cancer cell migration and adhesion. We studied how the Rho GTPases mediated SDF-1α effect, by demonstrating that RhoA and Rac1 were sequentially activated at different concentration of SDF-1α, thus, promoting different metastatic properties through the modulation of cancer cells phenotype.

## Methods

### Cell cultures

Breast cancer cell line MDA-MB231, MCF7, SK-BR-3, MDA-MB261, Hs578T, T47D was purchased from ATCC and cultured following ATCC recommendations (ATCC, Manassas, VA, USA). DMEM high glucose (Hyclone, Thermo Scientific), 10 % FBS (Hyclone, Thermo Scientific), 1 % Penicillin-Streptomycin-Amphotericyn B solution (Sigma), 1X Non-Essential Amino-Acid (Hyclone, Thermo Scientific) and 1 % L-glutamine. MDA-MB231 cell lines were stably transduced by lentiviral vectors encoding eGFP (Genethon, Evry). Bone Marrow host cells (BMHCs) are mesothelial cells extracted from bone marrow aspirates of donors within a bone marrow transplantation program in the Hematology Department of Hôtel-Dieu in Paris [32]. The samples were obtained with the approval of an appropriate ethics committee and are in compliance with the Helsinki Declaration. BMHCs were maintained and expanded in culture using DMEM low glucose (Hyclone, Thermo Scientific), 30 % FBS (Hyclone, Thermo Scientific), 1 % Penicillin-Streptomycin-Amphotericyn B solution (Sigma). All cultured cells were incubated as monolayers at 37 °C under a water-saturated 95 % air-5 % CO2 atmosphere and media are renewed every 2–3 days.

Bone marrow samples were obtained from the Hematology Department of Hôtel-Dieu in Paris. All necessary ethical approval for the collection and use of the tissue samples and cell lines were obtained. The Hotel Dieu IRB is the ethics committee who approved the bone marrow samples and reviewed the project. All donors were healthy donors in a bone marrow graft program and informed consent was given. Written informed consent for participation in the study was obtained from participants or, where participants are children, a parent or guardian. All samples obtained were de-identified.

### Tissue micro-array construction and immunohistochemistry

Immunohistochemistry was performed on 5-μm thick routinely processed paraffin sections. Using a tissue microarray instrument (Beecher Instruments, Alphelys™), we removed representative areas of the tumor from paraffin embedded tissue blocks. The antibodies were incubated for 30 or 60 min and then revealed by a system of polymers coupled to the peroxidase (EnVision™ kit, Dako Cytomation, Glostrup, Denmark).

### Cell proliferation assay

Cells were plated at 50,000 cells per well in a 6 well plate in medium without FBS. Cells were then counted with a hemocytometer for the following six days every two days. Two wells were counted per conditions. For co-cultures, only the green cells (MDA-GFP) were counted. The experiment was performed in triplicates.

### Confocal microscopy

Live-cell microscopy was used to analyze co-culture of mesothelial and tumor cells. Cells were labeled with 1 mg/ml Alexa FluorW 594 conjugated wheat germ agglutinin (WGA, Invitrogen SARL, Cergy Pontoise, France) at 5 μg/ml for 10 min at 37 °C in the dark. WGA is a probe for detecting glycoconjugates, which selectively binds to N-acetylglucosamine and Nacetylneuraminic acid residues of cell membranes. Confocal microscopy was performed on fixed cells in 3.7 % formaldehyde. Cells were stained with a 50 μg/ml AF647-conjugated phalloidin (Sigma) to label actin filaments. Slides were mounted in a mounting media SlowFade® Gold Antifade Reagent with DAPI (Invitrogen). Imaging was performed using a Zeiss confocal Laser Scanning Microscope 710 (Carl Zeiss). Post-acquisition image analysis was performed with Zeiss LSM Image Browser Version 4.2.0.121 (Carl Zeiss).

### Electron microscopy

Co-culture of MDA-MB231 and BMHC were established for 48 h. Cells were subsequently washed with PBS and fixed for 45 min in 30 % formaldehyde +5 % glutaraldehyde. Fixed cells were then centrifuged, treated with 50 mM ammonium chlorate, dehydrated and enveloped in Epoxy resin at low temperature at polymerization conditions. The micro sections (600–800 Au) were performed and colored with uranyl acetate and lead and visualized on a Philips CM 10 electron microscope as previously described [33].

### Motility assay in agarose gel

Our agarose gel assay was conceived based on the publication of Mousseau et al. [34]. First, we designed two molds using 15 ml tube lids, one with 3 lids allowing us to quantify the motility of the cells between a control wells, and a treated one and one with 5 lids for the competition experiments.

#### Agarose gel well formation

A 1 % solution of agarose was prepared in medium composed of 50 % phosphate-buffered saline (PBS) and 50 % DMEM (Gibco®; Invitrogen, Carlsbad, CA, USA) supplemented with 10 % heat-inactivated FBS and 2 mM L-glutamine (Invitrogen). For a 100-mm diameter Petri dish, 20 mL final agarose solution was needed. Type II agarose (Sigma-Aldrich) was added to PBS. After agarose was dissolved in PBS in a microwave oven, the solution was autoclaved and sterile DMEM was added. The agarose solution was poured into the Petri dish around the specific molds to give the well shape (Additional file 1: Figure S1). After 20–30 min of cooling, the gel was humidified with 5 mL DMEM, and the template was removed. Before performing the cell assay, 5 mL FBS-free DMEM were added to the gel for 1–6 h in order to stabilize the pH, for saturation of the gel and to prevent culture medium from diffusing in the gel during the experiment.

#### Chemotaxis assay and measurements during cell migration

Cells were seeded at a density of 80 000 cells per well in a complete medium with FBS. After 24 h, the medium was replaced with a starving medium with FBS. For the 3 wells experiments, the MDA-MB231 were seeded in the middle well, starving medium was poured as negative control on one side, on the other side BMHC or SDF-1α concentration tested was used. For the 5 wells experiments, MDA-MB231 were seeded on the middle well, one well was poured with starving medium as negative control and different concentrations of SDF-1α were added in the three other wells. Due to the short SDF1-α half-life, the medium was replaced every day [35]. Image capture and measurements were performed using an AMG Evos microscope (Fisher Scientific). The number of migrating cells was evaluated by measuring the distance traveled by the cells. The starting reference point used was the beginning of the agarose wall.

### Wound closure assay

Migration was assessed by wound closure assay as previously described [6]. Cells cultured at confluence in 24-well plates were scratched with a small tip along the ruler. Cells were then cultured for 24 h in starvation media with or without SDF-1α.

### Calcein-AM staining

For the calcein-AM assay, cells were prepared as previously described [36]. Briefly, cells were stained with 0,25 μM of calcein-AM. After 15 min incubation at 37 °C, cells were washed twice with PBS.

### Tube formation assay

A Matrigel-based capillary-genesis assay was performed on cells to assess their ability to form an organized tubular network as previously described [37]. Cells were starved for 6 h then 100,000 cells were cultured on 250 μl of Matrigel (BD bioscience). The degree of tube formation was quantified at different time-points by measuring the intersection of tubes in five randomly chosen fields from each well using ImageJ.

### Western blot analysis

Western blot were carried out as previously described [38]. Immunostaining was carried out using a goat monoclonal antibodies against RhoA (2117), Rock2 (9029), Rac1 (2465), Cdc42 (2466), SDF-1α (3740), integrin (α4-4600; α5-4705; αV-4711; β3-4702; β4-4707; β5-4708), actin (3200) (1/1000, Cell signaling) and a secondary polyclonal mouse anti-goat antibody HRP conjugated (1/2000, cell signaling). Blots were developed using HRP and chemiluminescent peroxidase substrate (#CPS1120, Sigma). Data were collected using Geliance CCD camera (Perkin Elmer), and analyzed using ImageJ software (NIH).

### Pulldown assay

Cells were treated as indicated with SDF-1α. Pulldown assays were performed according to the manufacturer's protocol (Rho activation assay kit 17–294 and Rac1 activation assay kit 17–441, both from Millipore, Billerica, MA).

### RT-PCR analysis

Total RNA was extracted from cells cultures using Trizol. After genomic DNA removal by DNase digestion (Turbo DNA free kit, Applied Biosystems), total RNA (1 μg) was reverse transcribed with oligodT (Promega) using the Superscript III First-Strand Synthesis SuperMix (Invitrogen). PCR analysis was performed as previously described [38] with a MasterCycler apparatus (Eppendorf) from 2 μL of cDNA using primers from IDT (Table 1).

**Table 1:** Primers Sequence used for RT-PCR

Primer	FORWARD	REVERSE
CXCR4	GCCTTATCCTGCCTGGTATTGTC	GCGAAGAAAGCCAGGATGAGGAT
SDF-1	ACTGGGTTTGTGATTGCCTCTGAAG	GGAACCTGAACCCCTGCTGTG
GAPDH	AGCCACATCGCTCAGACAC	GCCCAATACGACCAAATCC

### SiRNA treatment

siRNA against human RhoA (Santa Cruz biotechnology) were introduced into cells by lipid mediated transfection using siRNA transfection medium, reagent and duplex (Santa Cruz biotechnology) following manufacturer recommendations. Briefly the day before transfection cells were platted at 2,5 .10^5^ cells per well in 2 ml antibiotic-free normal growth medium supplemented with FBS. Cells were incubated until they reach 60–80 % confluence. The duplex solution containing the siRNA is then added to the cells. After 5 to 7 h, antibiotic are added in each well and the cells are incubated for 24 h more. The media is then replaced by normal growth media and cells are used for experiments and assay by RT-PCR to analyze the expression of RhoA gene.

### RNA silencing and generation of lentiviral particles

Stable lentiviral particles expressing small hairpin interfering RNAs (shRNA) targeting human Rac1 mRNA in MDA-MB231 cells were generated using cDNA lentiviral shRNA vector (MISSION® shRNA Plasmid DNA, Sigma Aldrich). The sequence was: 5′-CCGGCCTTCTTAACATCACTGTCTTCTCGAGAAGACAGTGATGTTAAGAAGGTTTTTG-3′. We used a scramble non-sense RNAi sequence with no homology in the mouse genome (shScramble) to control the unspecific effects of shRNA (Sigma Aldrich). In brief, 293 T cells were co-transfected with shRNA lentiviral plasmid or shScramble lentiviral plasmid plus the lentiviral packaging and envelope plasmids (Sigma Aldrich) using lipofectamin2000 and following manufacturer’s instructions. Medium containing generated viral particles was collected three days post transfection. Generated shRac1 lentiviral particles were used to infect MDA-MB231 cells using 4 μg/ml polybrene in order to generate stable shRac1 expressing cells. Puromycin selection (2 μg/ml) was used to select the infected cells.

### Adhesion assay

adhesion assay Tissue culture plates (96-well) were pre-coated with bone marrow host cells to reach 70 % confluency or with nonspecific attachment factors (Chemicon) following manufacturers’ instructions, or with human endothelial cells. MDA-MB-231 previously transfected with eGFP were seeded at 5 *10^4^/well in 200 ml serum-free medium, and allowed to attach for 1 h at 37 °C with BMHC. Non-adherent cells were removed by gentle washing with PBS. The adherent cells were quantified by quantifying the fluorescence at 560 nm in each well using a Wallac Flite fluorescence reader. In order to determine the role of the different GTPases in adhesion to stromal cells we used specific siRNA transfected MDA-MB-231.

### Flow cytometry

Fluorescence (FL) was quantified on a SORP FACSAria2 (BD Biosciences). Data were processed with FACS Diva 6.3 software (BD Biosciences) as previously described [39, 40].

### Statistical analysis

All quantitative data were expressed as mean ± standard error of the mean (SEM). Statistical analysis was performed with SigmaPlot 11 (Systat Software Inc., Chicago, IL). A Shapiro-Wilk normality test, with a p = 0.05 rejection value, was used to test normal distribution of data prior further analysis. All pairwise multiple comparisons were performed by one way ANOVA followed by Holm-Sidak posthoc tests for data with normal distribution or by Kruskal-Wallis analysis of variance on ranks followed by Tukey posthoc tests, in case of failed normality test. Paired comparisons were performed by Student’s t-tests or by Mann–Whitney rank sum tests in case of unequal variance or failed normality test. Statistical significance was accepted for p < 0.05 (*), p < 0.01 (**) or p < 0.001 (***). All experiments were performed in triplicates.

## Results

### Breast cancer cells interact with bone marrow host cells (BMHC)

**Fig. 1:**
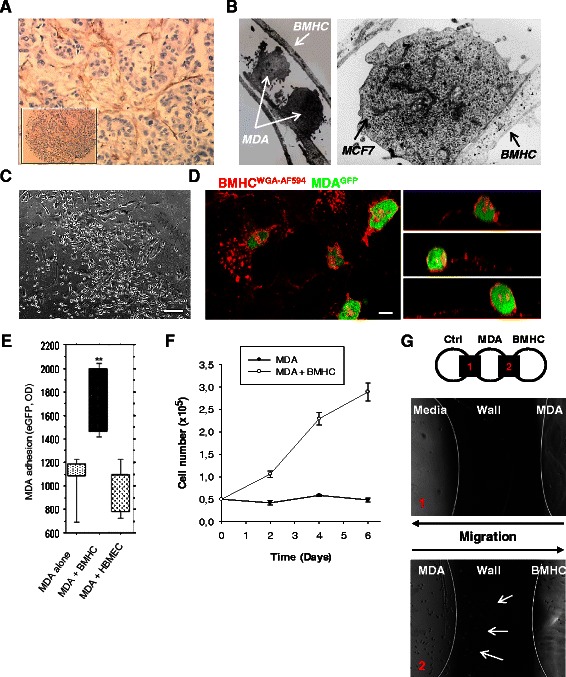
Intercellular interactions between cancer cells and Bone Marrow Host Cells (BMHC). **a** Paraffin-embedded immunohistochemistry. Antibody against CD-10 was used. Picture showed a network of BMHC (brown cells) surrounding cancer cell clusters (blue cells). The insert picture showed the metastatic node the tissue micro-array. **b** Electronic microscopy imaging. MDA-MB-231 and BMHC or MCF7 and BMHC were co-cultured during 48 h and analyzed by electronic microscopy. A pseudopodia of BMHC with two MDA-MB-231 cells were closely interacting with the pseudopodia (*left panel, arrows*). Very close interaction between the two cellular membranes of MCF7 and BMHC can be observed with formation of tight junction (*right panel, arrows*). **c** Co-culture of BMHC and MDA-MB231 in phase microscopy. Cancer cells are growing on BMHC. *Scale bar 250 μm*. **d** Confocal imaging of BMHC and eGFP MDA-MB231 co-culture. BMHC were co-cultured with tumor cells for 3 days. Before imaging by confocal microscopy, co-cultures were stained with Alexa Fluor 594 conjugated-wheat germ agglutinin (WGA). Z-X reconstitution shows that cancer cells (green) are growing on BMHC. *Scale bar 10 μm*. **e** Adhesion assay testing the specificity of the adhesion between MDA-MB231 cells and BMHC. BMHC were plated up to 60 % confluency, 50,000 eGFP MDA-MB231 were allowed to adhere for 1 h. HBMEC (human bone marrow endothelial cells) or plastic were used as negative control. **f** Proliferation assay. MDA-MB231 were plated and counted every 2 days in presence or not of BMHC during 6 days. BMHC were able to increase proliferation of MDA-MB231. **g** Migration in agarose gel assay. MDA-MB231 cells were seeded in the central well. Media only was poured in the left well as negative control and BMHC were seeded in the right well. Cells could be observed during migration through the agarose gel (black part, wall). The picture represents MDA-MB231 cells migration through the agarose wall to the BMHC well at day 4 (bottom picture, arrows) or to the media only (top picture)

Tumor stroma is a composed of multiple cell types; we have previously described [33] the infiltration of ovarian cancer tumors by BMHC (CD9^+^CD10^+^). Here using paraffin-embedded immunohistochemistry of primary breast cancer specimen we found a network of BMHC (CD9^+^CD10^+^) surrounding cancer cell clusters (Fig. 1a). Electron microscopy analysis of co-cultures of BMHC and MDA-MB231 or MCF7 displayed close interactions with formation of tight junctions (Fig. 1b). When the two cell types were seeded at the same time at a ratio of 1/1, breast cancer cells (BCC) attached preferentially on BMHC compare to plastic or matrigel as shown on phase contrast and selected (x-z) sections, obtained from confocal microscopy (Fig. 1c-d). Adhesion of BCC and BMHCs was stronger than spontaneous adhesion to culture plate or to other cell type HBMEC (Human Bone Marrow Endothelial Cells) (Fig. 1e). We then investigated the functional benefit of such interaction. MDA-MB231 co-cultured with BMHC in serum free cytokine free media displayed a proliferative advantage compared to controls (Fig. 1f). Finally, in order to test the ability of BMHC to attract MDA-MB231, we developed an agarose-based migration assay to evaluate the motility of BCC (Fig. 1g). With this method, BMHC secreted factors rather than the components of extracellular matrix surrogates (such as Matrigel) would be responsible for the migration observed. In this set–up MDA-MB231 displayed increased migration toward BMHC compare to control media.

### SDF-1α/CXCR4 regulates adhesion to BMHCs

**Fig. 2:**
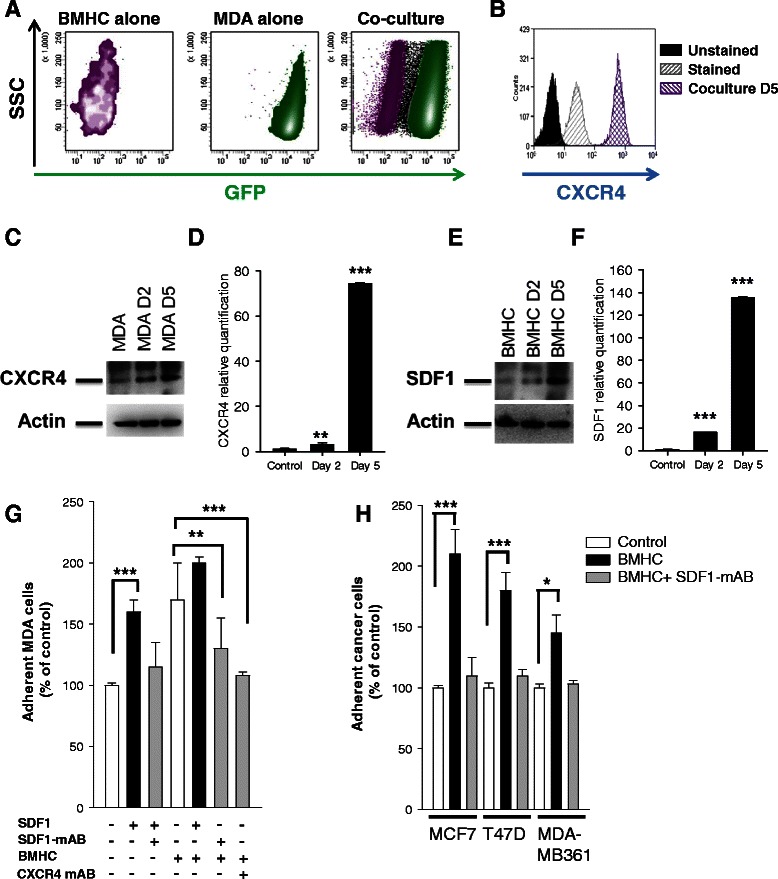
SDF-1alpha regulates interaction between MDA-MB231 and BMHC. **a** Flow cytometry cell sorting chart. MDA-MB231 (green) and BMHC (purple) were gated through GFP fluorescence intensity. **b** Flow cytometry analysis of CXCR4 expression. After 5 days of co-culture with BMHC, MDA-MB231 were cell sorted and stained for CXCR4. MDA-MB231 display an increase of the receptor after the co-culture. **c**-**d** MDA-MB231 after co-culture with BMHC. CXCR4 is increased in MDA-MB231 after 2 or 5 days of co-culture with BMHC in western blot (C) or real-time qPCR (D). Relative transcript levels are represented as the ratios between the 2 subpopulations of their 2^–ΔΔCp^ real-time PCR values. These are data representative of three different experiments. **e**-**f** BMHC after co-culture with MDA-MB231. SDF-1α is increased in BMHC after 2 or 5 days co-culture with MDA-MB231 in western blot (E) or real-time qPCR (F). CXCR4 is increased in MDA-MB231 after 2 or 5 days of co-culture with BMHC (right panel). **g** Adhesion assay. BMHC were plated up to 60 % confluency, 50,000 eGFP MDA-MB231 were allowed to adhere for 1 h in presence or not of SDF-1α and a SDF-1α or CXCR4 monoclonal antibody. Plastic was used as negative control. SDF-1α is involved in MDA-MB231 adhesion. **h** Adhesion assay. BMHC were plated up to 60 % confluency, 50,000 MCF7, T47D or MDA-MB361 (stained with Calcein-Am) were allowed to adhere for 1 h in presence or not of SDF-1α and a SDF-1α monoclonal antibody

SDF-1α/CXCR4 interactions regulate chemotaxis and homing of BCC to the BHMC [16]. To investigate whether SDF-1α/CXCR4 plays a role in the interaction between BMHC and MDA-MB231, we performed cell sorting after 2 and 5 days of co-culture (Fig. 2a) and showed an increase of CXCR4 in the MDA-MB231 (Fig. 2b-d). Concurrently, an increase of SDF-1α production by BMHC could be observed after co-culture (Fig. 2e-f). Western blot and Flow Cytometry analysis revealed the same profile in 3 other breast cancer cell lines (MDA-MB361, MCF7 and T47D) and an absence of expression of CXCR4 or an absence of increase of this receptor upon co-culture with BMHC in two other one (Hs578T and SK-BR-3; Additional file 1: Figure S1B-C).

The specific adhesion between MDA-MB231 and BMHC was significantly reduced with the monoclonal antibody against SDF-1α or CXCR4 but not significantly increased by SDF-1α treatment, suggesting that BMHC secreted SDF-1α already induced optimal adhesion (Fig. 2g). MDA-MB361, MCF7 and T47D cell lines showed also an increased adhesion to BMHC, and the monoclonal antibody against SDF-1α was able to reduce it (Fig. 2h).

### SDF-1α has a concentration dependent effect on MDA-MB231

We hypothesized that during the migratory process BCC are exposed to different concentrations of SDF-1α. Muller et al. established that the optimal migration/invasion of MDA-MB231 during SDF-1α treatment was obtained at 100 ng/ml with lower migration and invasion at low and high doses [16]. We selected 3 different concentrations of SDF-1α, 50, 100 and 200 ng/ml and investigated the dose dependent response for adhesion, migration, invasion or proliferation of MDA-MB231 cells.

**Fig. 3:**
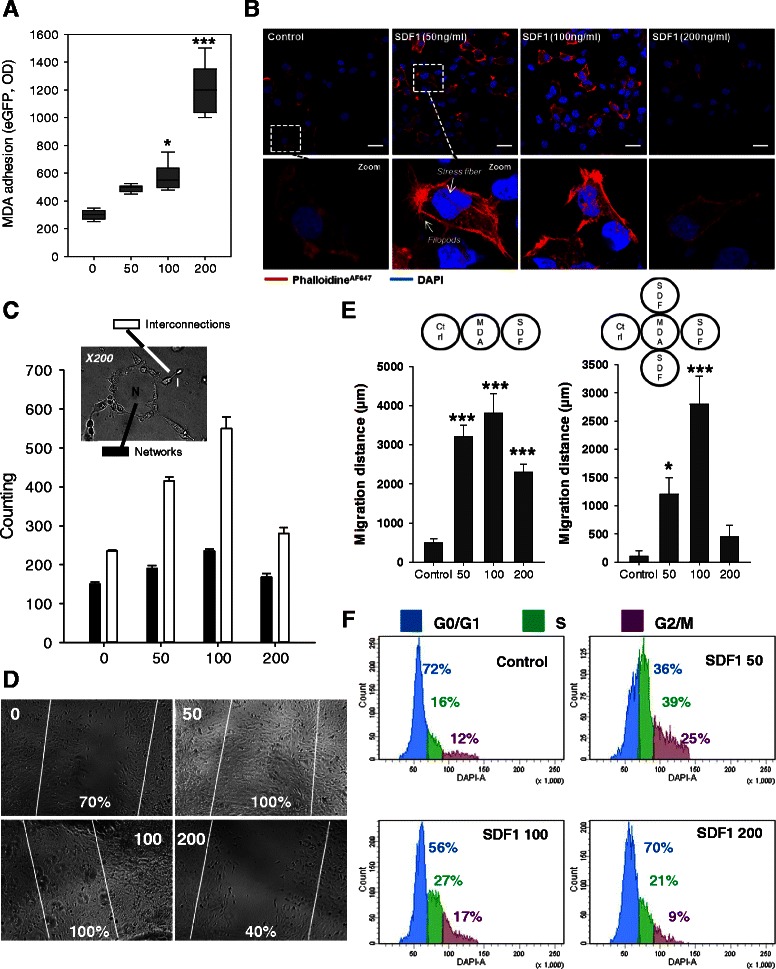
Differential effect of SDF-1alpha on MDA-MB231. **a** Adhesion assay testing the role of different concentration of SDF-1α. 50,000 eGFP MDA-MB231 were allowed to adhere for 1 h in presence or absence of SDF-1α (50, 100, 200 ng/ml). The maximum adhesion was observed at 200 ng/ml. **b** F-actin polymerisation in MDA-MB231. MDA-MB231 were grown on glass bottom slides with different concentration of SDF-1α (0, 50, 100 or 200 ng/ml) and actin cytosqueletton was revealed by a phalloïdin-fluorescein (1 μg/mL) labelling (red). More stress fiber and filipods can be seen (arrows) in MDA-MB231 treated with 50 or 100 ng/ml of SDF-1α. *Scale bar 20 μm.* **c** MDA-MB231 plasticity on Matrigel. MDA-MB231 were seeded on a 96-wells plate, coated with Matrigel in presence or absence of SDF-1α (50, 100 or 200 ng/ml). Microscopic pictures of cellular networks after SDF-1α stimulation were taken after 18 h of culture. Quantitative evaluation of the cellular interconnection (white) and network (black) are presented. The evaluation was made by counting on 10 different fields. The results are expressed as means with standard error. Interconnection and network number was increased when the cells are treated with 50 or 100 ng/ml of SDF-1α. **d** Wound closure assay. Migration ability of MDA-MB231 was tested after a scratch in presence of different concentration of SDF-1α (0, 50, 100 or 200 ng/ml). Only 50 and 100 ng/ml of SDF-1α enhanced MDA-MB231 motility. **e** Migration in agarose gel assay. MDA-MB231 cells were seeded in the central well. Media only was poured in the CTRL well as control and different concentration of SDF-1α were used in the right well for the 3 wells experiments (left panel) or simultaneity in the 5 wells experiments (right panel). Pictures were taken after 8 days and the distance travelled by the cells was calculated. The histograms present the results of 3 different experiments. **f** Cell cycle analysis. MDA-MB231 were treated with different concentration of SDF-1α (0, 50, 100 or 200 ng/ml) for 48 h and position in cell cycle were evaluated with NIM-DAPI by flow cytometry. 50 and 100 ng/ml of SDF-1α increased the number of cells in phase S (green) and G2/M (purple) and decreased the one in G0/G1 (blue). The results presents in this figure are representative of three different experiments

We demonstrated that maximal adhesion was obtained at a SDF-1α concentration of 200 ng/ml (Fig. 3a). Confocal microscopy imaging of MDA-MB231 treated with SDF-1α revealed an increase of F-actin staining in the periphery of the cells at 50 and 100 ng/ml (Fig. 3b). Stress fibers and filopods formation required for the invasion of malignant cells into tissues, were observed only at a concentration of 50 and 100 ng/ml. We then evaluated the role of SDF-1α in cellular plasticity by quantifying network formation on matrigel after 24 h of culture (Fig. 3c). Matrigel assays allow rapid quantification of the relative invasive potential of metastatic cells [41]. In this assay non tumorigenic cells generally do not grow; while low metastatic tumor cells form large round colonies, while high metastatic cells form branching invasive colonies [42]. SDF-1α at 50 and 100 ng/ml increased the formation of intercellular connections while the 200 ng/ml treatment resulted in decrease branching. In a wound-healing migration assay, 50 and 100 ng/ml of SDF-1α induced maximal migration (Fig. 3d).

To confirm this result, we developed an agarose gel assay to test the chemotactic properties of different concentration of SDF-1α (Fig. 3e left graph, Additional file 1: Figure S2). All concentrations of SDF-1α significantly attracted MDA-MB231 cells as compared to well with only media in it. In a 4 well setting, MDA-MB231 cells were more attracted toward 100 ng/ml of SDF-1α as compared to control and 50 or 200 ng/ml (Fig. 3e right graph, Additional file 1: Figure S3).

Finally SDF-1α treatment increased the number of cells in S and G2/M at 50 and 100 ng/ml (Fig. 3f). Altogether we confirmed the previously described role of SDF-1α on breast cancer migration and invasion. However, we also illustrated that high concentration of SDF-1α does not induce similar phenotypic modulation. As we verified that CXCR4 expression was not modified by high SDF-1α concentration (receptor endocytosis or down regulation leading to loss of effect) (Additional file 1: Figure S4A), we hypothesized that different downstream effectors could play a role in mediating the concentration dependent phenotypic modulation.

### SDF-1α mediated Rho GTPase and integrin regulation is concentration dependent

**Fig. 4:**
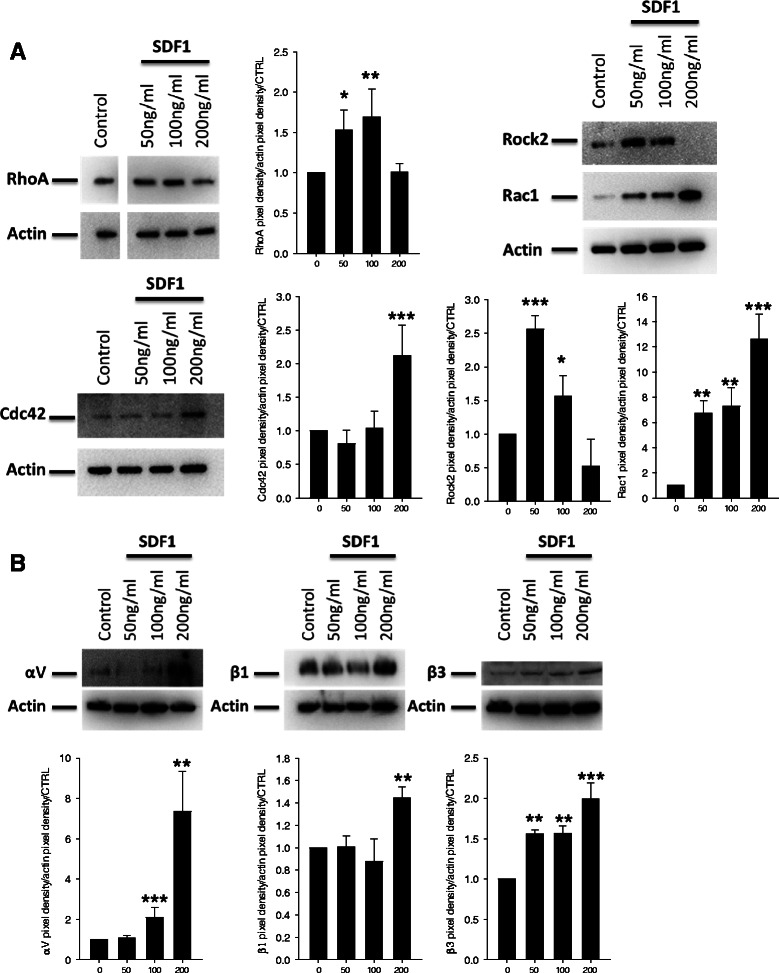
SDF-1alpha mediates Rho GTPase and integrin modulation. **a** Western blot analysis. MDA-MB-231 cells, serum-starved for 24 h, were treated with various concentration of SDF-1α (50, 100 and 200 ng/ml). Western blots against RhoA, Rock2, Rac1 and cdc42 were performed. The pixel density of each band has been divided by the corresponding actin band and by the control of the experiment. **b** Western blot analysis. MDA-MB-231 cells, serum-starved for 24 h, were treated with various concentration of SDF-1α (50, 100 and 200 ng/ml) for 4 h. Western blots against intergrin αV, β1 and β3 were performed. The pixel density of each band has been divided by the corresponding actin band and by the control of the experiment

Rho GTPases proteins are known to control the dynamics of the actin cytoskeleton during cell migration, proliferation or adhesion [24, 43]. To evaluate the impact of SDF-1α on regulation of these proteins, and upon the observation that different SDF-1α concentration induced different functional effects, MDA-MB231 cells were exposed to different concentration of SDF-1α (0, 50, 100 and 200 ng/ml). Western Blot showed an increase of RhoA and Rock2 protein up to a concentration of 100 ng/ml of SDF-1α (Fig. 4a). Interestingly, this effect was reversed when using 200 ng/ml of SDF-1α. Rac1 and CDC42 displayed a mirror profile with a maximum expression at a concentration of 200 ng/ml. We confirmed the same profiled of expression of RhoA and Rac1 upon SDF-1α in MCF7, T47D and MDA-MB-361(Additional file 1: Figure S4B-D).

As the changes in expression do not necessarily correlate with activation of Rho GTPases, we confirmed increased activation of RhoA and Rac1 using a GTP pull-down assay (Additional file 1: Figure S4E). Among the mediators of migration, invasion and adhesion integrin play a major role. The shift of integrin profile has been associated to the acquisition of a metastatic phenotype [44]. Thus we investigated their expression on MDA-MB231 after SDF-1α treatment (Fig. 4c and Additional file 1: Figure S5A). Western blot data show an up-regulation of αV, β1 and β3 protein after 4 h of stimulation with 200 ng/ml of SDF-1α. Moreover, using an inhibition strategy with monoclonal antibody, we were able to confirm the role of the αV, β1 and β3 integrin in the adhesion of MDA-MB231 (Additional file 1: Figure S5B).

### A balance between RhoA and Rac1 activation mediates differential effect of SDF-1α

**Fig. 5:**
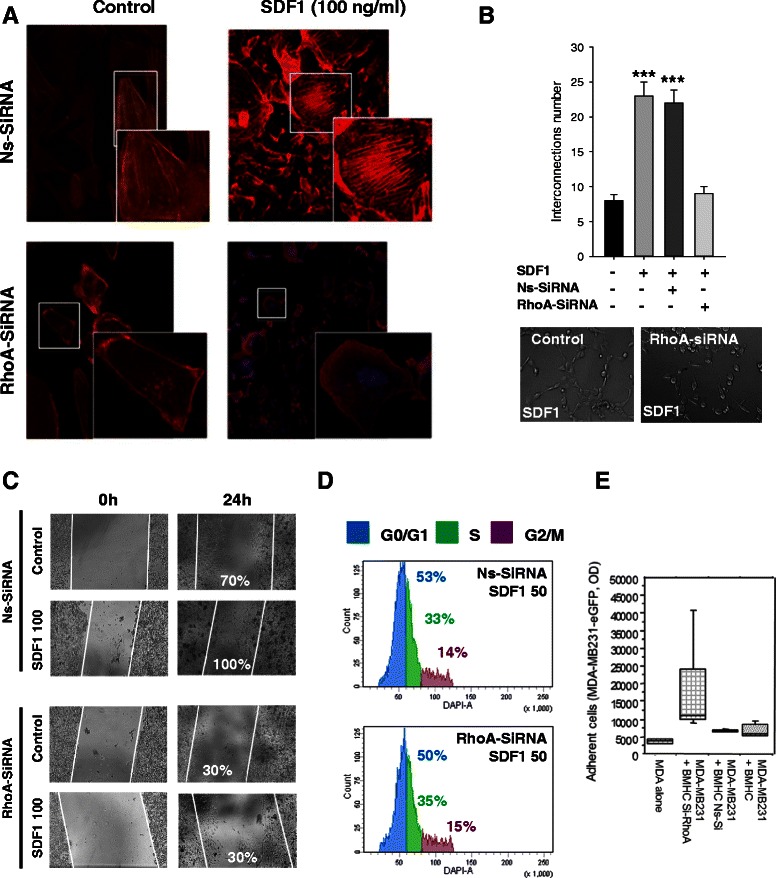
Functional consequences of inhibition of RhoA. **a** F-actin polymerisation in RhoA siRNA transfected MDA-MB231. Two days after si-RNA transfection, MDA-MB-231 were grown on glass bottom slides and actin cytosqueletton was revealed by a phalloïdin-fluorescein (1 μg/mL) labelling. Pictures present fluorescence microscope series of adherent MDA-MB-231 transfected with non-specific siRNA (ns-SiRNA), RhoA-specific (RhoA si-RNA) unstimulated or stimulated with SDF-1α (100 ng/mL). RhoA inhibition reverted the increased of stress fiber in the treated sample. **b** RhoA specific si-RNA transfected MDA-MB-231 plasticity on Matrigel. Two days after transfection, with non-specific si-RNA (ns-SiRNA) or RhoA specific (RhoA si-RNA) MDA-MB231 were seeded on a 96-wells plate, coated with Matrigel. Microscopic pictures of cellular networks after SDF-1α stimulation (100 ng/ml) were taken after 18 h of culture. Quantitative evaluation of the cellular interconnection is presented. The evaluation was made by counting the number of cellular interconnections on 10 different fields. RhoA inhibition reversed the interconnection number increase in the treated sample. **c** Wound Closure assay. Two days after transfection, with non-specific si-RNA (ns-SiRNA) or RhoA specific (RhoA si-RNA) MDA-MB231 migration ability was tested after a scratch with or without SDF-1α (100 ng/ml). RhoA inhibition supressed the effect of SDF-1α on MDA-MB231 motility. **d** Cell cycle analysis. Two days after transfection, with non-specific si-RNA (ns-SiRNA) or RhoA specific (RhoA si-RNA) MDA-MB231 were treated with or without SDF-1α (50 ng/ml) for 48 h and position in cell cycle were evaluated with NIM-DAPI by flow cytometry. The inhibition of RhoA doesn’t have any effect on the position of the cell cycle position of MDA-MB231. The results presents in this figure are representative of three different experiments. **e** Adherence of MDA-MB231 RhoA specific si-RNA transfected cells to BMHC. Stable eGFP-MDA-MB231 cells were seeded on the plate and allow to adhere for one hour. As displayed Si-RhoA transfected cancer cells displayed significantly increased adhesion compared to controls

**Fig. 6:**
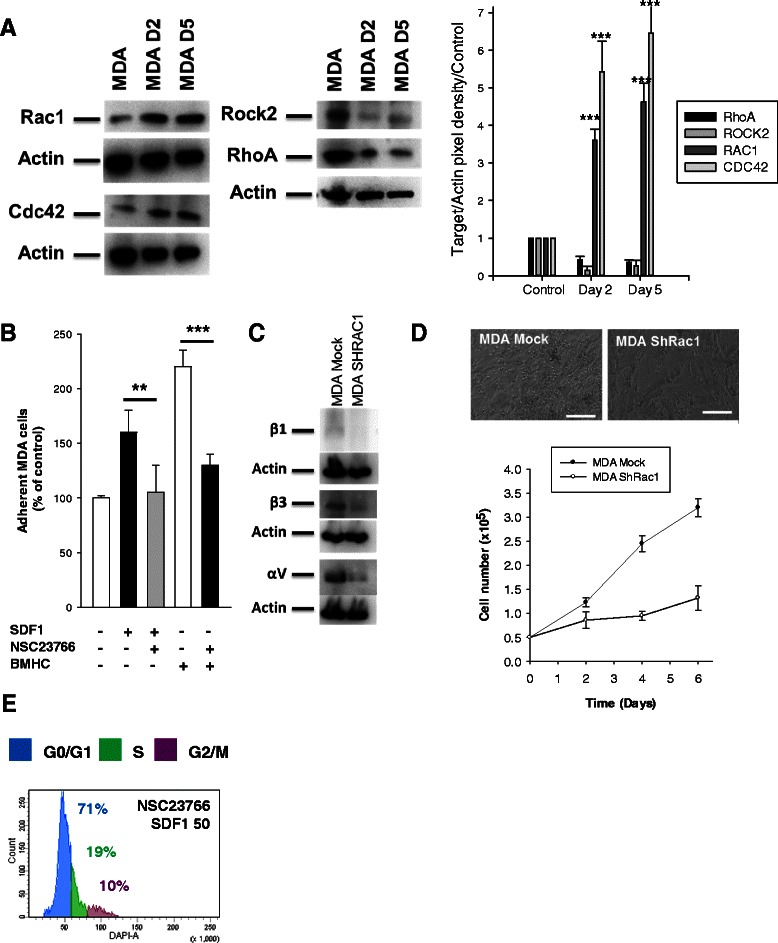
RhoGTPase modulation in a co-culture settings of MDA-MB231 and BMHC. **a** Western blot analysis. MDA-MB231 were sorted after a 2 days or 5 days of co-culture with BMHC as the chart presented in Fig. 2b. Co-culture increased the level of Rac1(up left panel) and Cdc42 (bottom left panel) but decreased Rhoa and Rock2 (middle panel) in MDA-MB231. The pixel density of each band has been divided by the corresponding actin band and then by the control of the experiment. The results are represented in the right histogram. **b** Adhesion assay. BMHC were plated up to 60 % confluency, 50,000 eGFP MDA-MB231 were allowed to adhere for 1 h in presence or not of SDF-1α and a Rac1 inhibitor (NSC23766). Plastic was used as negative control. Rac1 inhibition significantly decreased the adhesion of MDA-MB231 to BMHC. **c** Western blots analysis. MDA-MB-231 Mock or ShRac1, serum-starved for 24 h, were treated with SDF-1α (200 ng/ml) for 4 h. Western blots against integrin αV, β1, and β3 were performed. **d** Proliferation assay. MDA-MB231 Mock or ShRac1 were plated and counted every 2 days in presence of BMHC during 6 days in serum free condition. Images represent the co-culture of BMHC and MDA-MB231 Mock (left) or ShRac1 (right) in phase microscopy. *Scale bar 250 μm.* The chart represents the proliferation curve of MDA-MB231 Mock (black circle) or ShRac1 (white circle). BMHC were able to increase proliferation of MDA-MB231 Mock but not the ShRac1 one. **e** Cell cycle analysis. MDA-MB231 were treated with SDF-1α (50 ng/ml) and a Rac1 inhibitor (NSC23766) for 48 h and position in cell cycle were evaluated with NIM-DAPI by flow cytometry. The inhibition of Rac1 reversed the effect of SDF-1α on the cell cycle position of MDA-MB231

To confirm the essential role of both RhoA and Rac1 we used an inhibition strategy. Using a Si-RhoA (Additional file 1: Figure S5C), we were able to show reduced actin polymerization when MDA-MB231^RhoA-^ were treated with SDF-1α (Fig. 5a). The number of cellular extension was also decreased by the inhibition of RhoA (data not shown). SDF-1α mediated increase of intercellular connection was reversed in Si-RhoA transfected cells (Fig. 5b). The inhibition of RhoA has a drastic negative effect on the migration and proliferation of the MDA-MB231 (Fig. 5c and d). However adhesion to BMHC was increased in Si-RhoA transfected cells (Fig. 5e) suggesting that activation of RhoA has a negative effect on the MDA-MB231 binding to the BMHC. As a decrease in RhoA expression was leading to increased adhesion, we hypothesized that the balance between RhoA and Rac1 could be a mediator of the SDF-1α effect. We used the cell-sorting gate set-up in Fig. 2a to separate MDA-MB231 after a co-culture of 2 or 5 days with BMHC. The sorted cells displayed an increase of Rac1 and Cdc42, but a decrease of Rock2 and RhoA (Fig. 6a). Using NSC23766, a widely used inhibitor of Rac1 activation, we were able to demonstrate a decrease of MDA-MB231 adhesion to both plastic and BMHC despite SDF-1α treatment (Fig. 6b). We then generated a knock-down of Rac1 through ShRNA (Additional file 1: Figure S5C). We previously demonstrated an up-regulation of αV, β1 and β3 protein after 4 h of stimulation with 200 ng/ml of SDF-1α. Interestingly, when Rac1 was silenced in MDA-MB231, a 200 ng/ml of SDF-1α treatment didn’t lead to increased integrin expression confirming the major role of Rac1 in MDA-MB231 adhesion through integrin αV, β1 and β3 (Fig. 6c). When MDA-MB231 ShRac1 cells were co-cultured for 6 days with BMHC, we noticed a decrease in the number of cancer cells present on BMHC (Fig. 6d top panels). Moreover, MDA-MB231 ShRac1 cells co-cultured with BMHC in serum free cytokine free media didn’t display any proliferative advantage as compared to MDA-MB231 Mock (Fig. 6d bottom panel). Finally, we confirmed that Rac1 inhibition reduced the number of proliferating cells using a cell cycle analysis in presence of SDF-1α (Fig. 6e).

## Discussion

We demonstrated that the migration and adhesion sequences of breast cancer cells, induced by SDF-1α gradients, involves successively the activation and inactivation of RhoA and an increased expression of Rac1 through the gradient.

Krook et al. recently underlined the role of Rac1 and Cdc42 for the CXCR4 dependent metastasis of Ewing sarcoma cells to SDF-1α-rich microenvironments such as lungs and bone marrow [45]. Cytokine mediated tumor cell migration or chemo invasion, is an important early step in cancer metastasis. Muller et al. have shown that SDF-1α was mainly produced by organs that are frequent sites of breast cancer metastasis [16]. Experimental metastatic mouse models have shown that targeting or silencing CXCR4 inhibited development of metastasis in breast cancer [16, 46–49].

While the role of SDF-1α in the metastatic spread in solid tumors has been clearly established, its role in activation of RhoGTPases has only been described in the context of multiple myeloma where SDF-1α binding to its receptor CXCR4 induces chemotaxis and motility through RhoA activation [23].

However, it is essential to understand how a single cytokine can modulate apparently contradictory effects. The importance of cytokine gradients has been illustrated in the developmental context, where SDF-1α gradient is primordial during migration of the zebrafish posterior lateral line primordium [50]. Kim et al. have investigated the role of SDF-1α gradient and their data is concordant with our findings as they demonstrated reduction of MDA-MB231 velocity at high concentration of SDF-1α (above 150nM) [51]. Similarly, the migration of leukemic cell lines (KG-1v, KG-1a, HL-60, and leucapheresis-derived CD34^+^) was reduced at high concentration of SDF-1α (180 vs 60nM) [52].

Our main hypothesis is that breast cancer cells are not exposed to similar concentration of SDF-1α during the metastatic process. The differential tissue concentration of cytokines has been shown in different physiological and pathological contexts such as ischemia and tumor grade in glioblastoma [53, 54].

We have shown for example that endothelial cells from the bone marrow secrete a high concentration of SDF-1α as compared to endothelial cells from other organs [55]. Such differential organ concentration can influence cancer cell plasticity. Indeed extensive work from Massague clearly demonstrates that the microenvironment of the host organ plays a role in selecting specific cancer cell clones or phenotype. Interestingly in their data and among the genes involved in Bone Marrow metastasis, CXCR4 expression was significantly increased [56].

SDF-1α induced-RhoGTPases activation (expression) in cancer has been previously linked to cell migration. In our settings, CXCR4 expression was not modified with low and high concentration of SDF-1α. Hence, suggesting different mechanisms for the differential regulation of RhoA and Rac1 expression. The differential regulation of RhoA and Rac1 has been previously suggested, where by the expression of dominant negative Rho family GTPases mimics activation of other member of the Rho GTPases family [57]. Inactivation of Rac1 can result in an inversion of polarity associated to an activation of RhoA [58]. Metastatic cells interacting with bone marrow cells display higher levels of Rac1 *in vitro* and *in vivo* [59–62].

We found that SDF-1α concentration level radically modifies the integrin expression profile, where high SDF-1α concentration increased in αV, β1 and β3. αVβ3 integrin regulates Rac1 in endothelial migration and angiogenesis [63]. αVβ1 activates Rac1 in CHO cells and stop cell migration and increase adhesion through cell polarization [64]. Rac1 up regulation has been associated to RhoA inhibition and linked to the modulation of the cytoskeleton [65].

If the clinical relevance of our findings is confirmed, then one might think that targeting RhoA could induce increased adhesion and potential homing; down-regulating the Rac1 signaling would induce increase migratory proprieties. SDF-1α blockade is currently used in hematopoietic stem cell mobilization, and is under evaluation in the treatment of leukemia and solid tumors [66].

## Conclusion

**Fig. 7:**
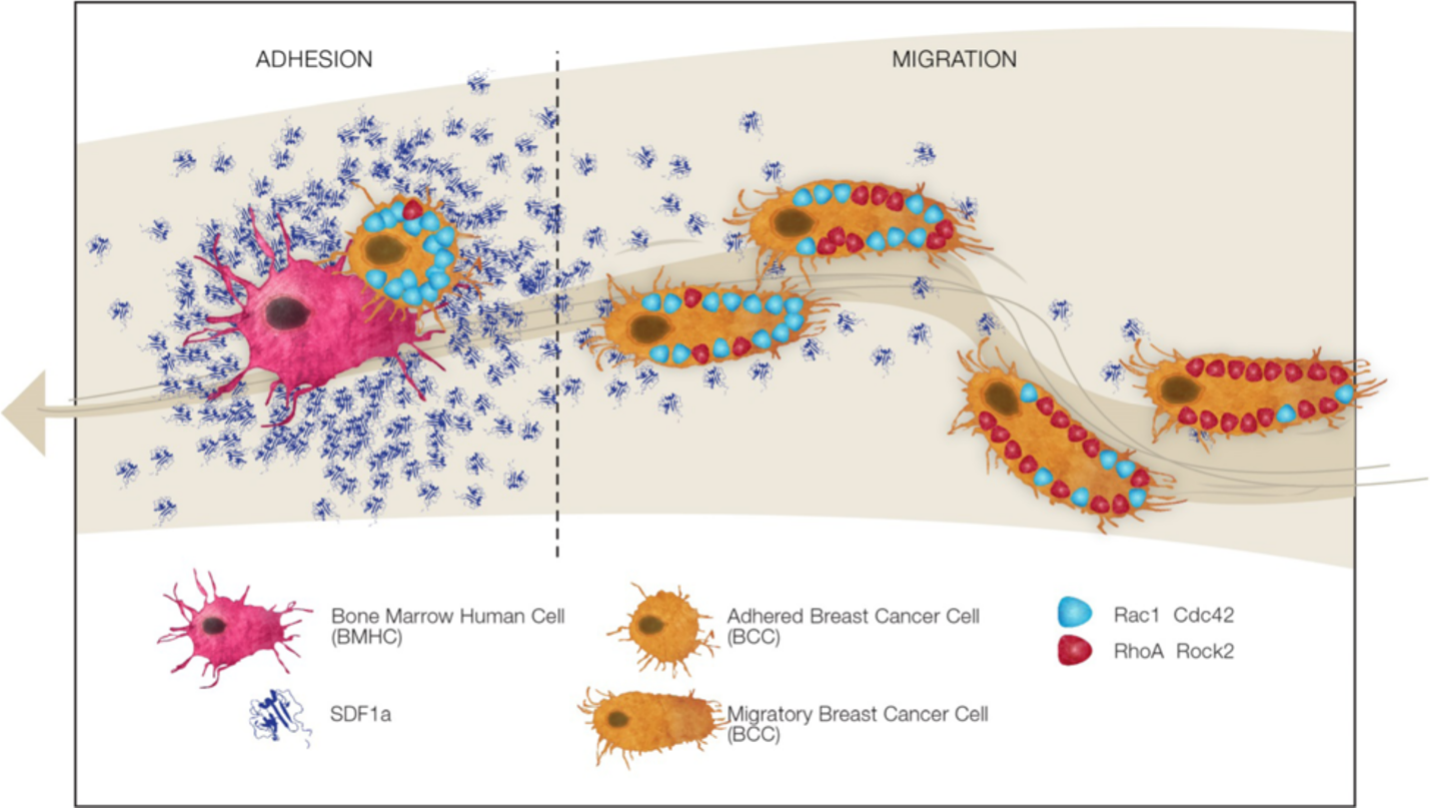
Differential role of small GTPase in BMHC and MDA-MB231 interactions

Our understanding of metastatic development in breast cancer is crucial to design novel therapeutic strategies. The role of the microenvironmental cues, in particular the cytokine mediated signaling has been already established in breast cancer metastasis. Here using an *in vitro* approach we were able to explain two apparently contradictory roles of the interaction between SDF-1α/CXCR4. We showed that while low concentration of SDF-1α promoted cell migration through RhoA activation, high concentration of the cytokine promoted intercellular interaction through Rac1 activation (Fig. 7). Our findings shed light on the dynamics of the interaction between breast cancer cells and their microenvironment, as well as the dual role of SDF-1α.

## Additional file

Additional file 1: Figure S1. **A.** This figure displays pictures of the 3 and 5wells agarose petri dish used for the migration assay. **B.** Western blot analysis of six different breast cancer cell lines, SK-BR-3, T47D, MDA-MB361, MDA-MB231, MCF7 and Hs578t for CXCR4 expression. **C.** Flow cytometry chart of CXCR4 expression in T47D, MDA-MB361, MCF7 or SK-BR-3 cell sorted after a co-culture of 5 days with BMHC. **Figure S2.** This figure displays representative pictures taken for the 3 wells agarose migration assay. **Figure S3.** This figure displays representative pictures taken for the 5 wells agarose migration assay. **Figure S4. A.** Flow cytometry against CXCR4. Plots for unstained and MDA-MB231 untreated were overlaid (left) and plots for MDA-MB231 treated with the different concentration of SDF-1α (right). **B-D.** Western blot analysis. T47D (B), MCF7 (C) or MDA-MB-361 (D) cells, serum-starved for 24 h, were treated with various concentration of SDF-1α (50, 100 and 200 ng/ml). Western blots against RhoA and Rac1 were performed. **E.** RhoA and Rac1 Activation Assay. SDF-1α treatment increased the amount of active GTP-bound RhoA (RhoA-GTP) and active GTP-bound Rac1 (Rac1-GTP). Total RhoA and Rac1 served as loading control (n = 3). **Figure S5. A.** Western blot analysis. MDA-MB-231 cells, serum-starved for 24 h, were treated with various concentration of SDF-1α (50, 100 and 200 ng/ml) for 4 h. Western blots against intergrin α4, α5, β4 and β5 were performed. **B.** Adhesion assay testing the role of integrin in the adhesion of MDA-MB231 cells under SDF-1α treatment. Fifty thousand eGFP MDA-MB231 were allowed to adhere for 1 h in presence or absence of monoclonal antibody against integrin β1, β3 or αV or a mix of the 3 antibodies. **C.** RhoA SiRNA efficiency in MDA-MB231. Five days after the SiRNA treatment, RhoA level was evaluated by Western Blot. RhoA is completely abolished in MDA-MB231 after SiRNA treatment. **D.** Rac1 ShRNA efficiency in MDA-MB231. Rac1 level was evaluated by Western Blot. (PDF 2821 kb)
