# Supplementary material for: SDF-1alpha concentration dependent modulation of RhoA and Rac1 modifies breast cancer and stromal cells interaction
Source: BMC Cancer. 2015 Aug 1;15:569. doi: 10.1186/s12885-015-1556-7 (PMC4522077; doi:10.1186/s12885-015-1556-7)

# Supplementary Figure 1

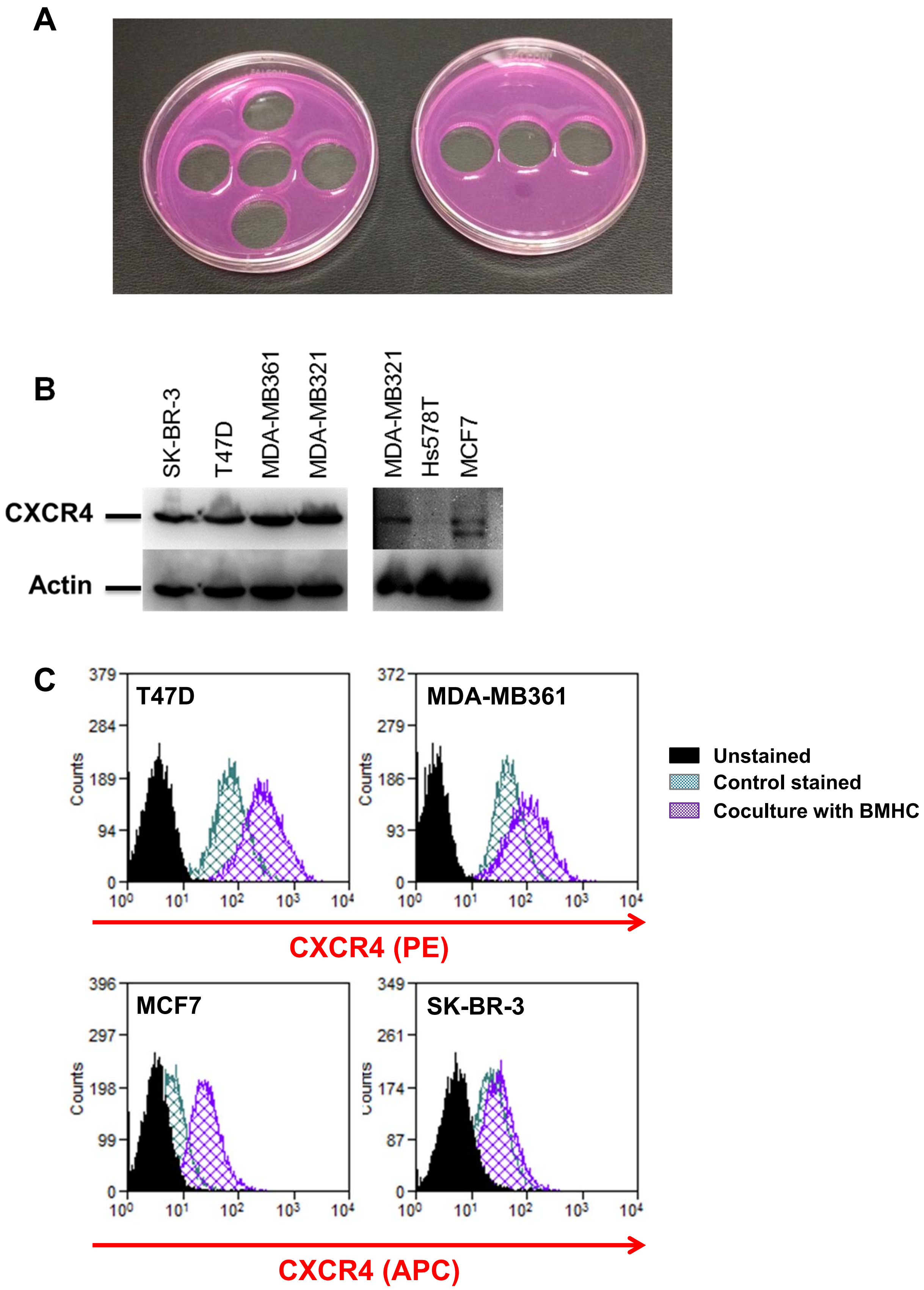

# Supplementary Figure 2

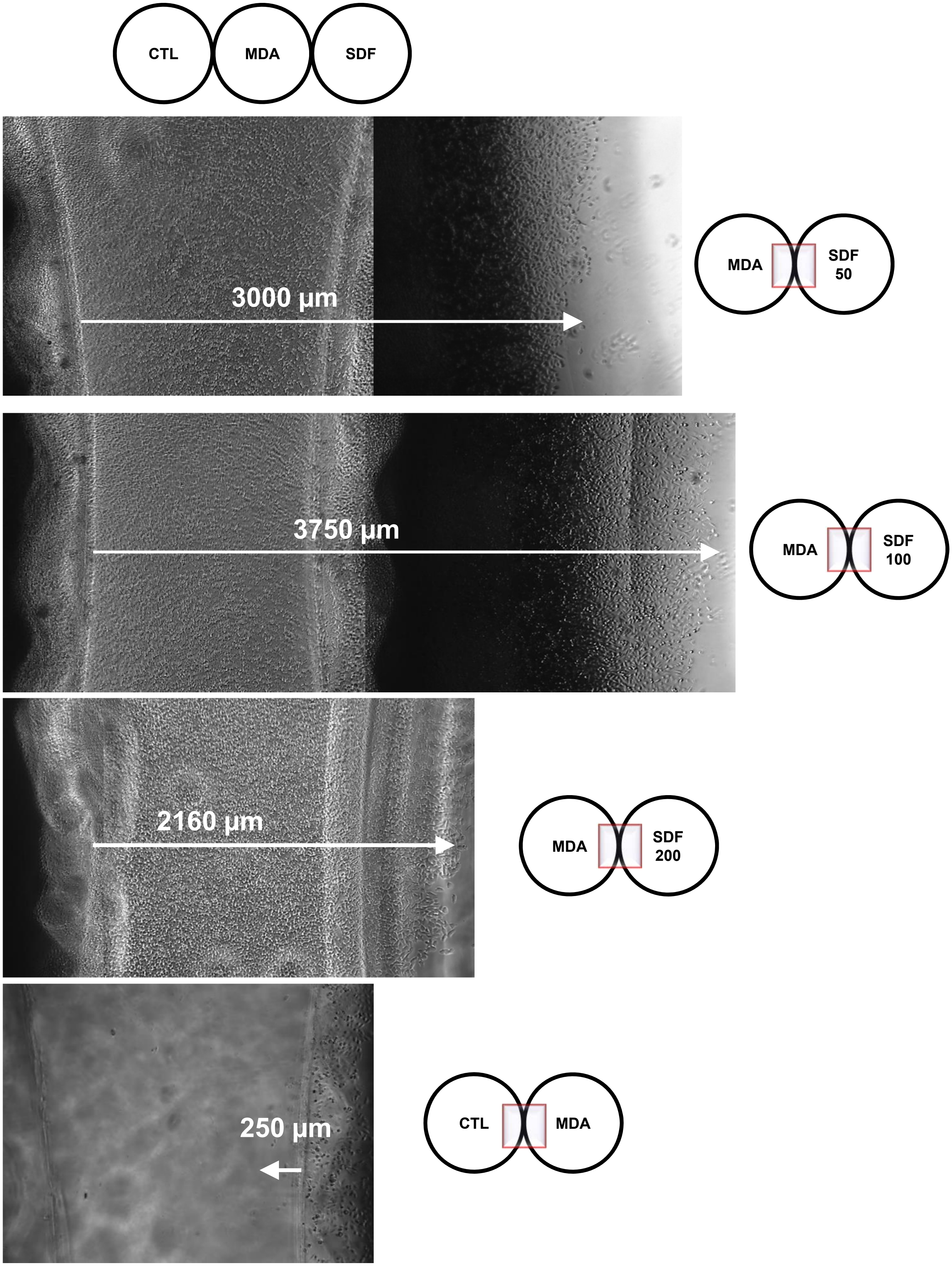

# Supplementary Figure 3

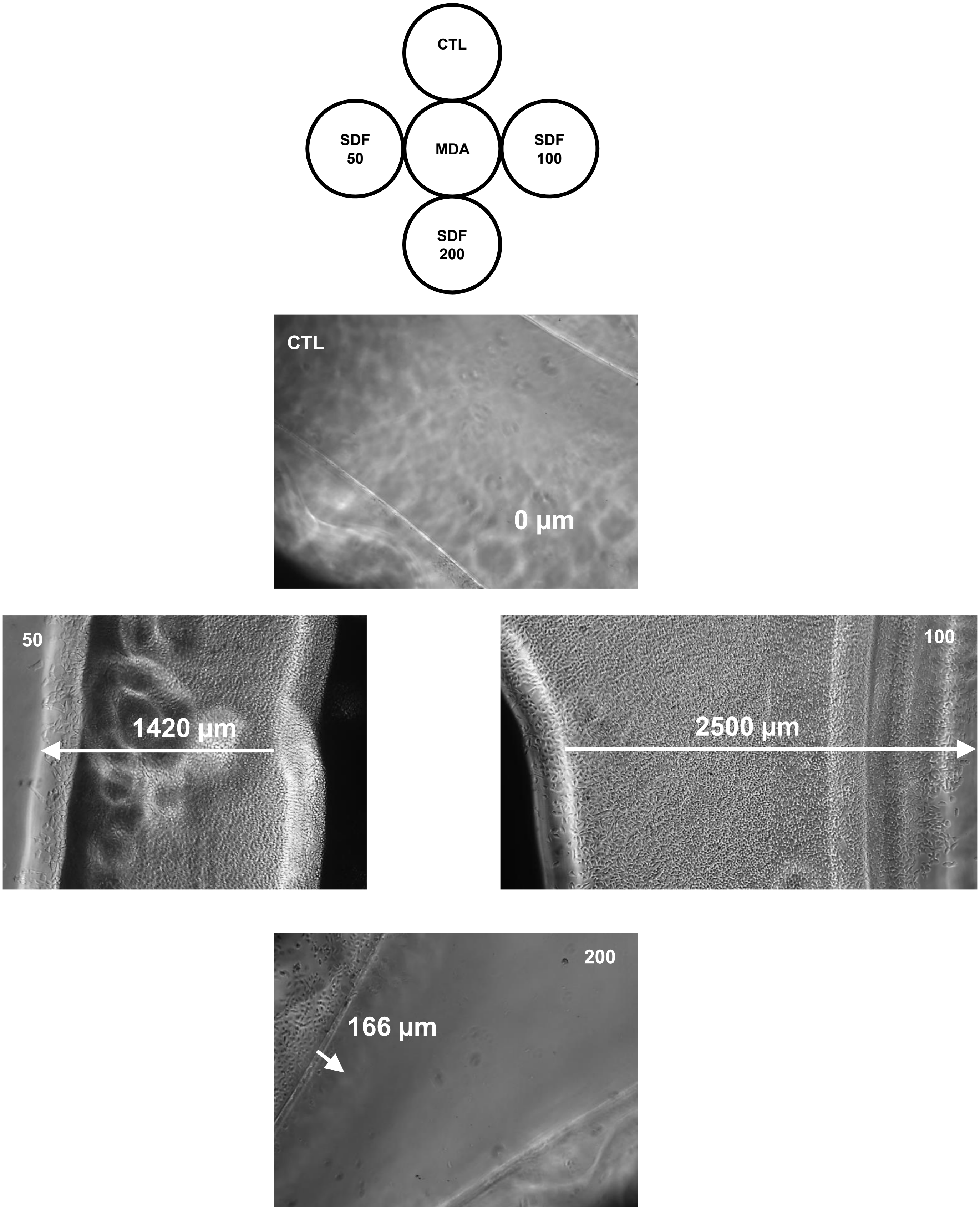

# Supplementary Figure 4

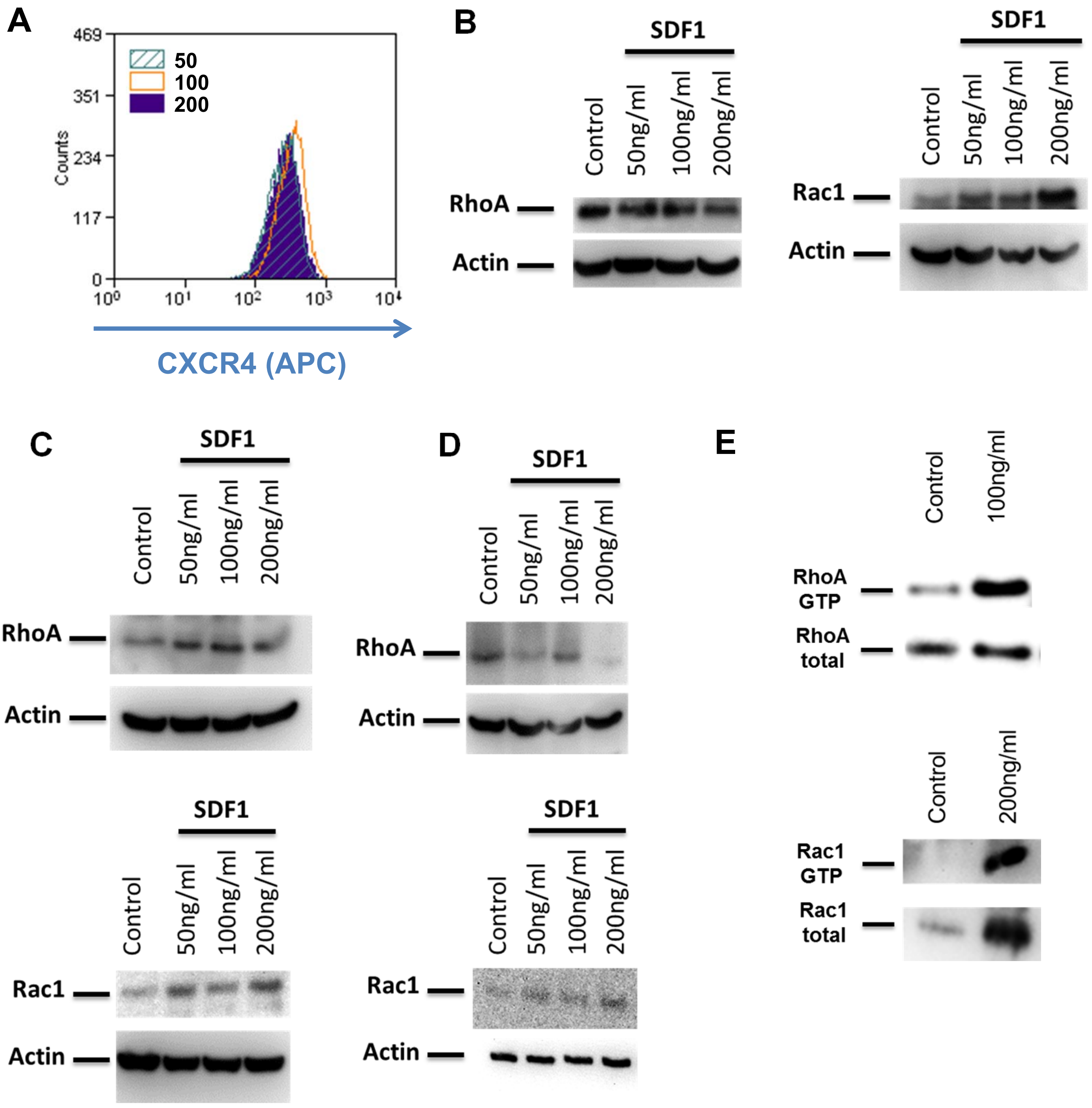

# Supplementary Figure 5

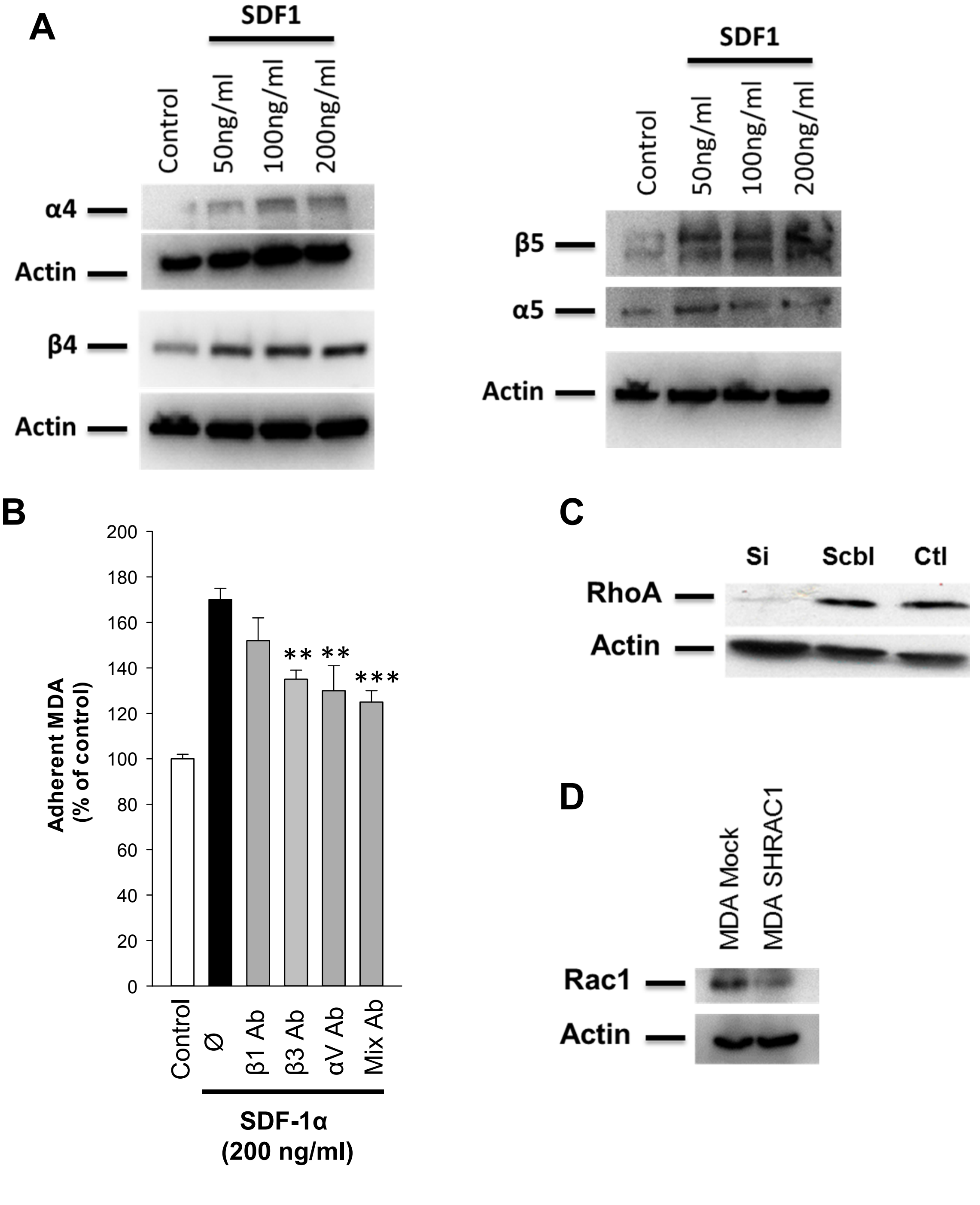

Supplement: Additional file 1: Figure S1. — A. This figure displays pictures of the 3 and 5wells agarose petri dish used for the migration assay. B. Western blot analysis of six different breast cancer cell lines, SK-BR-3, T47D, MDA-MB361, MDA-MB231, MCF7 and Hs578t for CXCR4 expression. C. Flow cytometry chart of CXCR4 expression in T47D, MDA-MB361, MCF7 or SK-BR-3 cell sorted after a co-culture of 5 days with BMHC. Figure S2. This figure displays representative pictures taken for the 3 wells agarose migration assay. Figure S3. This figure displays representative pictures taken for the 5 wells agarose migration assay. Figure S4. A. Flow cytometry against CXCR4. Plots for unstained and MDA-MB231 untreated were overlaid (left) and plots for MDA-MB231 treated with the different concentration of SDF-1α (right). B-D. Western blot analysis. T47D (B), MCF7 (C) or MDA-MB-361 (D) cells, serum-starved for 24 h, were treated with various concentration of SDF-1α (50, 100 and 200 ng/ml). Western blots against RhoA and Rac1 were performed. E. RhoA and Rac1 Activation Assay. SDF-1α treatment increased the amount of active GTP-bound RhoA (RhoA-GTP) and active GTP-bound Rac1 (Rac1-GTP). Total RhoA and Rac1 served as loading control (n = 3). Figure S5. A. Western blot analysis. MDA-MB-231 cells, serum-starved for 24 h, were treated with various concentration of SDF-1α (50, 100 and 200 ng/ml) for 4 h. Western blots against intergrin α4, α5, β4 and β5 were performed. B. Adhesion assay testing the role of integrin in the adhesion of MDA-MB231 cells under SDF-1α treatment. Fifty thousand eGFP MDA-MB231 were allowed to adhere for 1 h in presence or absence of monoclonal antibody against integrin β1, β3 or αV or a mix of the 3 antibodies. C. RhoA SiRNA efficiency in MDA-MB231. Five days after the SiRNA treatment, RhoA level was evaluated by Western Blot. RhoA is completely abolished in MDA-MB231 after SiRNA treatment. D. Rac1 ShRNA efficiency in MDA-MB231. Rac1 level was evaluated by Western Blot. (PDF 2821 kb) [file 12885_2015_1556_MOESM1_ESM.pdf]
